# Are we missing opportunities? Physiotherapy and physical activity promotion: a cross-sectional survey

**DOI:** 10.1186/s13102-017-0084-y

**Published:** 2017-11-29

**Authors:** Nicole Freene, Sophie Cools, Bernie Bissett

**Affiliations:** Faculty of Health, University of Canberra, Canberra, 2601 Australia

**Keywords:** Physical activity, Health promotion, Survey

## Abstract

**Background:**

Physical activity (PA) promotion in healthcare is an important strategy for increasing PA levels. Physiotherapists are well-positioned to promote PA, however no studies have investigated PA promotion by physiotherapists Australia-wide.

**Methods:**

An online survey of practicing Australian physiotherapists was conducted to investigate knowledge of the Australian Physical Activity and Sedentary Behaviour (PASB) guidelines and factors associated with increased promotion frequency. Participants were asked to state the PASB guidelines and a 4-component scoring system was used to measure knowledge. Multivariate logistic regression analysis was conducted to assess factors associated with frequency of promotion.

**Results:**

257 Australian physiotherapists completed the survey. Only 10% were able to accurately state the PASB guidelines and 54% reported promoting PA to 10 or more patients per month. Males were nearly three times more likely than females to promote PA to 10 or more patients per month (OR 2.68, 95% CI 1.25–5.74). Those who lacked counselling skills and felt PA promotion wouldn’t change their patients’ behaviour were much less likely to promote PA.

**Conclusion:**

Australian physiotherapists have poor knowledge of the Australian PASB guidelines and infrequently promote PA. Education and training in PA counselling and behaviour change strategies is indicated to enhance PA promotion by Australian physiotherapists.

## Background

The World Confederation of Physical Therapists states that physiotherapists are ideally positioned to deliver physical activity (PA) promotion for both primary and secondary prevention of non-communicable diseases (NCDs) [1]. They have training and experience in educating clients and in prescribing PA programs for a range of NCDs, such as cardiovascular conditions and diabetes [1–5]. Physiotherapists often work with individuals over a period of weeks and months, providing them with the opportunity to follow-up on behaviour change strategies [1]. Despite this, there is little evidence of PA promotion by physiotherapists globally [6].

Physiotherapists around the world perceive that PA promotion is part of their role, with 50–99% of physiotherapists from Australia, Belgium, Brazil, Ireland and Nigeria agreeing this is part of their normal clinical work [5, 7–11]. A high proportion of physiotherapists report they are confident delivering general PA advice (90–97%) [5, 7, 10]. However, there is some evidence that physiotherapists may feel less confident promoting PA to individuals with specific health conditions, such as individuals with respiratory and cardiovascular conditions [11]. Lack of consultation time is the most commonly perceived barrier for PA promotion [5, 9, 10, 12]. In contrast to this, physiotherapists worldwide agree that brief education and advice is the most feasible option for PA promotion [5, 7, 9, 10, 12].

Reported frequency of PA promotion amongst physiotherapists is fair, with 36–54% of physiotherapists in Australia and Nigeria promoting PA beyond therapeutic exercise to 10 or more patients per month [5, 10]. Additionally, knowledge of the PA guidelines appears to be poor. Shirley et al. (2010) found that only one third of physiotherapists in New South Wales (NSW), Australia were aware of the Australian PA guidelines. Physiotherapists in Ireland and Belgium were found to have slightly better knowledge of their countries’ PA guidelines (51–53%) [7, 8]. Studies have found that PA promotion by physiotherapists can increase PA levels and reduce sedentary behaviour in the short (12 weeks) and long-term (2 years) [13, 14]. Yet, physiotherapists internationally do not seem to be taking advantage of opportunities and promoting PA to their patients [6].

One strategy to increase the population’s PA levels is for health practitioners to incorporate PA promotion into routine healthcare [15]. To enable integration of PA promotion into routine physiotherapy care, it is essential to understand the current state of physiotherapists’ PA promotion and knowledge across a number of settings, including private practices, hospitals and community settings. The aim of this study is to describe Australian physiotherapists’ knowledge of the Australian physical activity and sedentary behaviour (PASB) guidelines, and factors that are associated with greater frequency of PA promotion.

## Methods

### Study design

A cross-sectional online survey was undertaken between January and March 2016. Using voluntary sampling, physiotherapists working in Australia were recruited through advertisements placed in physiotherapy-specific electronic publications and communications and via professional networks. Participants were included in the study if they self-reported they were currently practicing within Australia as a physiotherapist in any setting, including private practice, community health, public hospital or private hospital. To increase response rates, participants who completed the online questionnaire had an opportunity to win one of four AUS$100 sports vouchers.

### Survey

The online questionnaire was based on a previous questionnaire that had been used in physiotherapy and General Practice populations [5, 16]. Minor adaptations were made to make it applicable to all settings, with the questionnaire taking approximately 10 min to complete. Participants were asked about their PA promotion practices, perceived role in PA promotion, barriers to promotion, feasibility of different promotion strategies, their knowledge of the PA public health messages and their personal PA levels. Most items were scored using a five-point Likert scale. The questionnaire also included general demographic questions, such as age, gender and details of the participant’s work (average number of patients seen each week, number of years in practice, usual number of hours worked each week, workplace postal code and the type of practice). To facilitate analysis and reporting, each of the multiple response option variables were later transformed into a binary variable. For example, agree (combining the 2 ‘agree’ options) versus disagree (combining ‘neutral’ with the 2 ‘disagree’ options). Finally, participants were asked if they were aware of Australia’s PASB Guidelines for Adults (18–64 years) [17] and, if so, to describe these. Each description was assessed for accuracy using a four-component scoring system. The four components of the guidelines assessed were duration, intensity, resistance exercise and minimising sedentary behaviour. To achieve a complete correct answer participants must have mentioned all four components according to the Australian PASB guidelines for Adults (18–64 years), that is, accumulate 150 to 300 min of moderate intensity physical activity or 75 to 150 min of vigorous intensity physical activity or a combination of both each week; muscle strengthening exercise at least 2 days per week; and minimise time spent in prolonged sitting [17].

### Sample size

Sample size was based on the total number of registered physiotherapists in Australia in March 2015 (*n* = 27,370) [18]. Using an online survey sample size calculator, with a confidence interval of 95% and a 5% margin of error, the sample size required was 379.

### Data analysis

Descriptive analyses were completed. Responses of physiotherapists who more frequently counselled patients to increase their PA levels (beyond therapeutic exercise) were compared to those who less frequently counselled patients to determine factors that are associated with greater frequency of PA promotion. This was achieved by dividing the total sample into two groups, ‘counselled <10 patients per month (less often)’ versus ‘counselled ≥10 patients per month (more often)’ [5, 10]. Associations between all variables were assessed using Spearman’s rho. Logistic regression analysis with manual backward stepwise elimination was utilized, commencing with all potential predictor variables to identify the most parsimonious model for predicting counselling patients more often compared to counselling patients less often to have a more PA lifestyle. The criterion of *p* < 0.05 was used to determine which variables were retained. The model was assessed for goodness of fit using the Hosmer-Lemeshow chi-square test and an area under the receiver operator characteristic (ROC) curve. All quantitative analyses were conducted using the Statistical Package for Social Sciences (SPSS) version 22. Significance level was set at *p* < 0.05.

## Results

A total of 257 Australian physiotherapists completed the survey (Table 1). The majority of participants were female and one third were less than 35 years of age. Nearly half of participants worked in private practice and almost one quarter worked in public hospitals. Of the 29 participants who reported ‘other’ kinds of practice, most reported working in a mix of practices, aged care or rehabilitation settings. Complete data sets were found in 98% of cases.

**Table 1:** Participant characteristics and characteristics of all registered physiotherapists in Australia

Characteristic	Participants (*n* = 257)	All registered Physiotherapists in Australia (*n* = 28,669)^a^
Age (years), n (%)		
< 35	93 (36)	13,641 (48)
35–44	48 (19)	6638 (23)
45–54	68 (26)	4557 (16)
> 54	48 (19)	3833 (13)
Gender, n (%)		
Male	57 (22)	9142 (31)
Female	200 (78)	19,527 (68)
Kind of practice, n (%)		
Private practice	114 (44)	–
Community health	39 (15)	–
Public hospital	62 (24)	–
Private hospital	13 (5)	–
Other	29 (11)	–
Number of years in practice, mean (SD)	18.1 (12)	–
Average number of patients seen per week, mean (SD)	41.8 (32)	–
Usual number of hours worked per week, mean (SD)	31.6 (13)	–
Australian workplace postcode, n (%)		
ACT	39 (15)	540 (2)
NSW	59 (23)	8352 (29)
VIC	62 (24)	7026 (25)
QLD	42 (17)	5299 (19)
SA	26 (10)	2283 (8)
WA	27 (11)	3454 (12)
TAS	0 (0)	448 (2)
NT	0 (0)	165 (1)
Encouraged a physically active lifestyle		
≥ 10 patients in the last month, n (%)	142 (55)	–
How physically active do you think you are currently compared with other Australians of your sex and age? (agree = more active), n (%)	203 (79)	–

- Data unavailable for total population of registered Physiotherapists in Australia

^a^Physiotherapy Board of Australia Registrant Data, March 2015 [18]

**Fig. 1:**
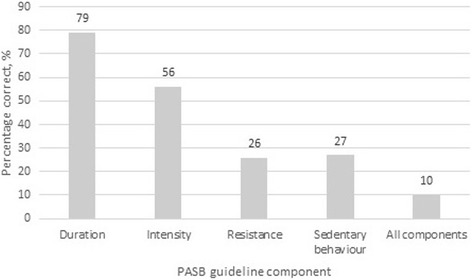
Knowledge of the Australian Physical Activity and Sedentary Behaviour (PASB) guidelines among Australian physiotherapists

Only one half of participants reported promoting PA beyond therapeutic exercise to 10 or more patients per month (Table 1). Ten percent of those who reported being aware of the Australian PASB guidelines for Adults (18–64 years) were able to accurately state all four components of the guidelines (Fig. 1). The majority of participants were able to accurately state the duration and intensity components but only one quarter of participants included the resistance or sedentary behaviour components in their responses.

Participants’ knowledge of the Australian public health messages varied (Table 2). The majority of participants agreed that several short walks of 10 min on most days is better than one round of golf per week for good health. Approximately half of participants agreed that generally being more active each day is enough PA to improve health. Less than half of participants agreed that half an hour of walking on most days is all the exercise needed for good health; and that exercise must make you puff and pant to be good for health.

**Table 2:** Australian physiotherapists’ knowledge, role perception, confidence, feasibility and barriers to physical activity promotion

Variable (n = 257)	n Agree (%)
Aware of the Australian PASB guidelines	168 (65)
Knowledge of PA message	
Taking the stairs at work and generally being more active each day is enough PA to improve health	138 (54)
Half an hour of walking on most days is all the exercise that is needed for good health	111 (43)
Exercise that is good for health must make you puff and pant	82 (32)
Several short walks of 10 min each on most days is better than one round of golf per week for good health	201 (78)
Health Professionals Role	
Discussing the benefits of a physically active lifestyle with patients is part of the health professionals role	254 (98.8)
Suggesting to patients ways to increase daily PA is part of the health professionals role	253 (98.4)
Health professionals should be physically active to act as a role model for their patients	250 (97.3)
Confidence in giving PA message	
I would feel confident in giving general advice to patients on a physically active lifestyle	245 (95.3)
I would feel confident in suggesting specific physical activity	239 (93.0)
Feasibility of PA promotion strategies	
Brief counselling integrated into regular consultations	241 (93.8)
Separate one-on-one consultations	135 (52.5)
Group sessions	173 (67.3)
Distribution of resources (such as brochures)	240 (93.4)
Barriers to PA promotion	
Lack of time	35 (13.6)
Lack of counselling skills	16 (6.2)
Lack of remuneration for promoting PA	14 (5.4)
Lack of interest in promoting PA	3 (1.2)
Feeling it would not change the patient’s behaviour	24 (9.3)
Feeling it would not be beneficial for the patient	2 (0.8)
Other	47 (18.3)

*PA* physical activity

The majority of participants agreed that PA promotion is part of the physiotherapist’s role and felt confident giving both general and specific PA advice to patients (Table 2). Overall, participants perceived few barriers to promotion (Table 2). ‘Other’ barriers were the most frequently reported, followed by lack of time. The most common ‘other’ barrier was PA promotion being inappropriate with some patients (e.g. low level of cognition, bed bound, acute unstable medical conditions), followed by feeling patients were not receptive. When considering feasibility of PA promotion strategies, brief counselling integrated into regular consultations was considered the most feasible strategy for promotion (Table 2).

Male participants were almost 3 times more likely to promote PA beyond therapeutic exercise to 10 or more patients per month than females. If participants agreed that separate one-on-one consultations were a feasible option for promotion they were 3 times more likely to promote PA to 10 or more patients per month. Table 3 details the results of the logistic regression modelling for factors associated with frequency of PA promotion by Australian physiotherapists. Participants who reported lack of counselling skills as a barrier to promotion were 83% less likely to promote PA to 10 or more patients per month. Those who reported feeling it wouldn’t change the patient’s behaviour were 73% less likely to promote PA to 10 or more patients per month. Treating a higher average number of patients per week was also a significant predictor of increased promotion frequency but number of hours worked per week was not retained in the model. The model demonstrated a good fit to the data and explained 33.2% of the variability.

**Table 3:** Logistic regression model for factors associated with frequency of physical activity promotion beyond therapeutic exercise by Australian physiotherapists

Variable	Odds ratio	95% CI
Barriers to promotion ^a^		
Lack of counselling skills	0.165*	0.038–0.710
Feeling it would not change the patient’s behaviour	0.271*	0.093–0.795
Feasible strategies for promotion ^a^		
Separate one-on-one consultations	3.228**	1.813–5.749
Average number of patients seen per week	1.028**	1.014–1.043
Gender ^b^	2.679*	1.250–5.740
Hosmer-Lemeshow goodness of fit (*p*)	0.593	
Nagalkerke R Square	0.332	
Area under ROC curve	0.803	

^a^reference agree ^b^reference male * *p* < 0.05 **p < 0.001

## Discussion

Australian physiotherapists have poor knowledge of the Australian PASB guidelines. Yet, the majority of participants felt confident promoting PA and agreed that PA promotion is part of their role, with half the participants promoting PA beyond therapeutic exercise to 10 or more patients per month. To our knowledge, this study is the first to find an association between gender and frequency of PA promotion, with male physiotherapists nearly three times more likely than females to promote PA to 10 or more patients per month, regardless of the number of hours worked per week. This finding may have important practical implications, with nearly 70% of Australian physiotherapists being female [18]. It is possible that this finding reflects a genuine disparity between male and female physiotherapists’ PA promotion behaviours, or alternatively may reflect a gender discrepancy in the self-reporting of behaviour. A recent survey of physiotherapists in Nigeria, found that female physiotherapists were assessing the PA levels of their patients more frequently than males [9]. This is the only other study to identify a link between gender and PA promotion by physiotherapists and, as it explored PA assessment rather than promotion, it is difficult to compare these results with those of our study. Future studies in this area should specifically explore gender factors to ascertain whether this relationship between PA promotion and gender is consistent across different samples.

Few studies have explored physiotherapists’ knowledge of the current PA guidelines. In 2010, Shirley et al. found that only one third of physiotherapists in NSW Australia were aware of the Australian PA guidelines. However, it was unclear if, and how, this study assessed the accuracy of participants’ responses when they were asked to describe the PA guidelines. In Ireland, 51% of physiotherapists were able to accurately recall the Irish PA guidelines [7]. A recent study of Belgian physiotherapists, assessing knowledge of the Belgian PA guidelines using a scoring system similar to that used here, reported that 53% of Belgian participants correctly mentioned 3 out of 4 components of the Belgian PA guidelines, with no report of the percentage of participants who could accurately state each individual component [8]. Whilst we found that the knowledge of the PA guidelines was much lower (10%) relative to previous studies, others did not require participants to include the recommendations for resistance exercise and/or sedentary behaviour in their responses. This is likely due to the sedentary behaviour component of the guidelines recently being added in Australia [19] and not yet being included in the Irish or Belgian guidelines [20, 21]. Nonetheless, Australian physiotherapists’ knowledge of the guidelines appears to be poor overall.

In addition to poor knowledge of the PA guidelines, participants’ knowledge of Australian public health messages relating to PA was limited. Only half of the participants agreed that generally being more active each day is enough PA to improve health. This may be due to physiotherapists believing that a larger amount of PA is needed for good health. This may also indicate that physiotherapists lack knowledge of the significant health benefits that can occur with small amounts of PA, such as half an hour of brisk walking on most days of the week [19]. Furthermore, it is possible that if physiotherapists don’t agree with these public health messages they may not promote them to patients, or they may advise patients that a larger amount of PA is necessary to achieve good health, making increasing PA levels seem less achievable for patients. One third of participants agreed that exercise that is good for health must make you puff and pant. This may also indicate a limited understanding that at least moderate intensity PA is recommended to achieve health benefits, or it may indicate a poor understanding of the definition of moderate intensity [17, 22, 23].

Regardless of the reasons why their knowledge was limited, our findings indicate that Australian physiotherapists require further education on all components of the Australian PASB guidelines, with a particular focus on resistance activities and sedentary behaviour. A systematic review of factors influencing health professionals PA promotion found that decreased PA awareness and knowledge results in decreased PA promotion [24]. Thus, PA awareness and knowledge appears to be essential for PA promotion, and this lack of PA knowledge is potentially rectifiable through education [25].

In our study, physiotherapists who lacked counselling skills, or felt that PA promotion wouldn’t change the patient’s behaviour and didn’t see one-on-one consultations as feasible options for PA promotion, were significantly less likely to promote PA. Previous studies have shown similar results [5, 24]. To reduce these barriers to PA promotion, physiotherapists may require further education and training in PA counselling and behaviour change strategies. However, it is important to consider that only 6% of all participants reported lack of counselling skills as a barrier to promotion. While this is an important area to address, it is not a common barrier reported by Australian physiotherapists.

The findings for PA promotion role perception, barriers, feasibility and confidence are similar to other studies conducted with physiotherapists around the world [5, 7–11]. Australian physiotherapists strongly agreed that PA promotion was part of their role and felt very confident that they could provide both general and specific PA advice to patients. This is in contrast to Mohan et al. (2012) that found Irish senior physiotherapists were less confident giving specific advice to individuals with respiratory and cardiovascular conditions. Importantly, here we found confidence was not significantly associated with frequency of promotion. Thus, confidence may not translate into PA promotion practice.

Barriers to PA promotion were few. ‘Other’ barriers, such as feeling it wouldn’t be appropriate with some patients, alongside lack of time, were the most frequently cited. While PA promotion may be inappropriate with some patients, such as those who are bed-bound or those with acute medical conditions, PA promotion can be adapted to be safe and appropriate for those with cognitive disorders and comorbidities [26–31]. Brief counselling integrated into regular consultations was found to be the most feasible option for PA promotion, which has also been found in previous studies [5, 10]. There is evidence that brief PA advice in primary care settings is effective [32]. Considering this, incorporating brief PA advice into routine physiotherapy consultations may be an achievable and successful PA promotion strategy.

Limitations to this study include a limited sample size and only offering the survey online, which may have limited responses from those that did not have access to an electronic device. Selection bias may have also occurred due to voluntary sampling, with the survey being promoted more widely in the area where the research team was located, and physiotherapists more interested in PA potentially more likely to complete the survey. There is also the potential for recall and social desirability bias. Physiotherapists may have over or under-estimated the number of patients they promoted PA to during the past month, and they may have inaccurately estimated their personal level of PA.

## Conclusion

Australian physiotherapists perceive PA promotion to be part of their role and feel confident promoting PA to their patients. Despite this, PA knowledge amongst Australian physiotherapists is poor. Training in brief PA counselling and behaviour change for practicing physiotherapists is indicated, particularly targeting female physiotherapists. If physiotherapists increase their PA knowledge, improve their PA counselling skills and take advantage of opportunities within routine physiotherapy consultations, they have the potential to increase PA levels of the Australian population.

## Abbreviations

NCDs: non-communicable diseases
NSW: New South Wales
PA: physical activity
PASB: physical activity and sedentary behaviour
